# Disparities in the offer of COVID-19 vaccination to migrants and non-migrants in Norway: a cross sectional survey study

**DOI:** 10.1186/s12889-022-13687-8

**Published:** 2022-07-04

**Authors:** Esperanza Diaz, Jessica Dimka, Svenn-Erik Mamelund

**Affiliations:** 1 Pandemic Center, Department for Global Public Health and Primary Care, University of Bergen, Bergen, Norway; 2 Norwegian Institute Public Health, Oslo, Norway; 3 Centre for Research On Pandemics & Society (PANSOC), Oslo Metropolitan University, Oslo, Norway

**Keywords:** COVID-19, Migrant, Migrant health, Health disparities, Vaccination

## Abstract

**Background:**

Vaccination is key to reducing the spread and impacts of COVID-19 and other infectious diseases. Migrants, compared to majority populations, tend to have lower vaccination rates, as well as higher infection disease burdens. Previous studies have tried to understand these disparities based on factors such as misinformation, vaccine hesitancy or medical mistrust. However, the necessary precondition of receiving, or recognizing receipt, of an offer to get a vaccine must also be considered.

**Methods:**

We conducted a web-based survey in six parishes in Oslo that have a high proportion of migrant residents and were hard-hit during the COVID-19 pandemic. Logistic regression analyses were conducted to investigate differences in reporting being offered the COVID-19 vaccine based on migrant status. Different models controlling for vaccination prioritization variables (age, underlying health conditions, and health-related jobs), socioeconomic and demographic variables, and variables specific to migrant status (language spoken at home and years lived in Norway) were conducted.

**Results:**

Responses from 5,442 participants (response rate of 9.1%) were included in analyses. The sample included 1,284 (23.6%) migrants. Fewer migrants than non-migrants reported receiving a vaccine offer (68.1% vs. 81.1%), and this difference was significant after controlling for prioritization variables (OR 0.65, 95% CI: 0.52–0.82). Subsequent models showed higher odds ratios for reporting having been offered the vaccine for females, and lower odds ratios for those with university education. There were few to no significant differences based on language spoken at home, or among birth countries compared to each other. Duration of residence emerged as an important explanatory variable, as migrants who had lived in Norway for fewer than 15 years were less likely to report offer of a vaccine.

**Conclusion:**

Results were consistent with studies that show disparities between non-migrants and migrants in actual vaccine uptake. While differences in receiving an offer cannot fully explain disparities in vaccination rates, our analyses suggest that receiving, or recognizing and understanding, an offer does play a role. Issues related to duration of residence, such as inclusion in population and health registries and health and digital literacy, should be addressed by policymakers and health services organizers.

**Supplementary Information:**

The online version contains supplementary material available at 10.1186/s12889-022-13687-8.

## Background

With approximately 530 million reported cases and more than 6 million reported deaths in over two years, the COVID-19 pandemic is the largest global public health crisis the living generation has experienced [1]. Although the pandemic is not over, vaccines have dramatically reduced its impacts [2]. However, this intervention is dependent on the majority of people, nationally and globally, having access to the existing vaccines [3]. Migrants, who are globally overrepresented in the COVID-19 statistics in high-income countries, have historically lower rates of vaccine uptake for several diseases [4–6], and the same is true for COVID-19 [7–9]. Although much effort has been made in Norway and other European countries to inform migrant groups about the vaccines and the importance of being vaccinated [10, 11], the proportion of vaccinated migrants remains lower than for the majority population [9]. This persistent difference suggests there are more complex causes than the availability of general information. Therefore, it is necessary to study other preconditions that must be in place for an individual to have an equitable chance of becoming vaccinated. Unravelling the underlying factors to explain differences between migrants and non-migrants is key to better health for all [12].

**Fig. 1:**
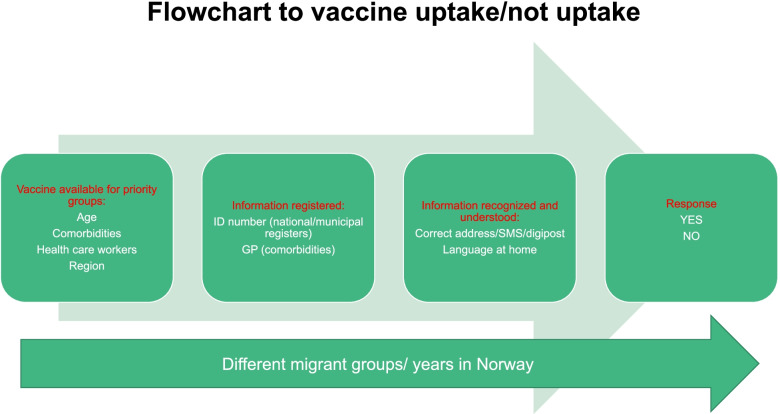
Steps to vaccine uptake at the time of the survey in Oslo

As shown in Fig. 1, when the vaccines first were available at the end of December 2020, some of the necessary preconditions in Norway to becoming vaccinated were 1) belonging to one of the priority groups, 2) being registered as such and 3) being able to recognize and understand the offer of a vaccine when it arrived in Norwegian. These preconditions might differ for different groups of migrants and non-migrants and change depending on the length of stay of a person in Norway and a given municipality. During the spring of 2021 before vaccines were more widely available, priority was given to individuals depending on age and comorbidities. Invitations to be vaccinated were sent based on the age registered in the population registers of the municipalities. In addition, information registered with general practitioners (GPs) on comorbidities was used by either the GPs directly or the municipality to contact adults at higher risk of developing serious COVID-19 disease regardless of age. The offer of a vaccine was sent in Norwegian by text messages (SMS), digital post or by letter for those who did not have digital post or did not open it after some days. Telephone calls were also used before the digital solution was in place. In addition, health care workers in direct contact with patients were prioritized and invited through their workplaces. The criteria used for vaccination roll-out did not explicitly differentiate between Norwegian-born and migrant residents. On March 9, 2021, however, the vaccine distribution strategy changed to increase availability in different regions proportional to the local burden of disease. Six parishes with high disease rates on the Eastern side of Oslo coincided with those where a higher proportion of migrants live, and the results we present in this paper rely on a data coming from a survey we carried out in these six parishes.

Levesque et al. [13] suggest a multidimensional conceptual framework of healthcare access that takes into account the population’s social determinants of health, resulting in five abilities needed by individuals and populations to be able to use the health care system: to perceive, to seek, to reach, to pay, and to engage in healthcare. Several studies have reported lower vaccination rates among migrants, and most authors have tried to understand the reasons why migrants and ethnic minorities do not get vaccinated in terms of misinformation, vaccine hesitancy, discrimination or medical mistrust [7, 14–19]. Although all these factors contribute to lower vaccination rates, they do not cover one of the necessary steps for a person to get vaccinated: the perception, this is to say the receipt and identification, of an offer. Furthermore, to our knowledge, no study to date has examined the degree to which migrants report receiving an offer to be vaccinated compared to the majority population.

Based on survey data from six parishes in Eastern Oslo with high proportions of migrants that were prioritized at the time of the survey, we aim to answer the following questions: Do migrants (from different groups) report receiving an offer for vaccination to a lesser degree than non-migrants? If so, which factors explain the differences?

## Methods

### Survey design

We conducted a web-based survey among residents living in six of the parishes of the capital city of Oslo, which was the epicenter of the COVID-19 pandemic in Norway, and that were prioritized for vaccines after 12 March 2021. The survey was developed in a collaboration between the Centre for Research on Pandemics & Society (PANSOC) at Oslo Metropolitan University and the Pandemic Centre at the University of Bergen. The survey consisted of closed-choice Likert-scale items, multiple answer questions and some open-ended questions on topics including disease burden, uptake of non-pharmaceutical interventions and vaccines, potential risk factors, and demographic variables. The variables used in this study are: 1) COVID-19 vaccine offer and uptake, 2) potential medical risk factors, such as non-communicable diseases, 3) socioeconomic and demographic questions including age, sex, and parish in Oslo, highest completed education, and type of job (phrased to capture potential exposure to COVID-19 at work), and 4) whether the respondent was a migrant to Norway, language spoken at home, birth country, and years in Norway. Supplementary Table 1 lists the survey questions and potential responses included in different analyses, as well as how we further modified or collapsed different categories.

### Target population and data collection procedure

The survey was carried out by Kantar on behalf of the researchers. Kantar is a professional survey administrator with access to a population database with all phone numbers in Norway, delivered by DataFactory (equivalent to “1881”, or the “White Pages” in the US). Text (SMS) messages in both Norwegian and English were sent to 59,978 potential adult (18 + years old) participants, sampled proportionally relative to population sizes for the six selected parishes. As our goal was to address questions related to migrant status, we targeted six eastern parishes of Oslo where many migrants live (Alna, Bjerke, Gamle Oslo, Grorud, Søndre Nordstrand, and Stovner). Recipients could click on a web link in the SMS which would take them to a start page for the survey. The information on the start page was given in in Norwegian but with a “For English, click here”-button with the same information given in English. On the start page of the survey participants were provided with the aim of the survey, expected time to finish (10 min), information on confidentiality and data handling, the researcher’s ethics approvals, and contact information to Kantar and the researchers. After selection of preferred language, (Norwegian, English, Arabic, Polish, Somali, and Urdu), participants were asked to consent to the survey. Informed consent was obtained from all participants. Participants were eligible for a drawing for three gift cards valued at NOK 1000 (approximately 100 Euros) each. Responses were collected between 16 and 24 June 2021, as the experience of Kantar is that there are few answers to be gathered after one week; this was also the case in this study. The survey was written in Norwegian and translated into English by the research team and subsequently professionally translated into Arabic, Polish, Somali, and Urdu, to encourage responses from migrants from the most populous groups in these areas who may not have responded to a Norwegian-language survey. However, more than 90% of respondents completed the survey in Norwegian.

### The sample

Although more than 10,000 SMS recipients began the survey, 5,447 surveys were completed for a response rate of 9.1% (5,447/59,978). Responses from five participants were removed during cleaning due to concerns about rapid completion speed, many skipped questions and/or nonsensical answers to open-ended questions; the final sample used in subsequent analyses was thus 5,442 respondents. Supplementary Table 2 provides a demographic breakdown of the net response rates and composition of the net and gross samples. Net response rates were somewhat higher for females, increased with age, and varied slightly by parish.

This paper focuses on differences based on migrant background, defined as being born abroad. Therefore, we limited our comparisons to individuals who reported being born outside of Norway, regardless of their parents’ birthplace, and those born in Norway to Norwegian-born parents, excluding the 507 (9.3%) of the net sample who reported being Norwegian-born children of migrant parents and 55 (1.0%) who did not report sufficient information about their own or their parents’ birth countries to determine status. The resulting sample was 4,880, with 1,284 (26.3%) migrants. In comparison, out of the full population of the six parishes, the percentage of migrants is estimated to be 34.5% [20].

### Hypothesis and analyses

Our main questions address whether there were potential differences in reporting being offered the COVID-19 vaccination based on migrant background. Therefore, after controlling for prioritization variables (age group in categories of 18–29, 30–44, 45–59, and 60 + years, underlying health conditions, and job type with healthcare workers with patient contact being given priority), there should theoretically be no differences in the report of being offered a vaccine between migrants and non-migrants. We tested this hypothesis using logistic regression. Additional regression models investigated the influence of a) other socioeconomic and demographic variables, and b) responses to questions that potentially reflect the experience as a migrant in Norway (language spoken at home, and length of stay in Norway measured as being less than versus equal or above the median of 15 years). Acknowledging the heterogeneity among migrants and potential roles of cultural or political issues and/or vaccination uptake in nearby countries instead of in Norway, different combinations of country of birth were also considered. Responses were grouped as: a) Nordic vs. non-Nordic, and b) EU vs. non-EU. Further, the top five most represented countries in the survey were also investigated separately. For all analyses, differences are considered statistically significant at the 0.05 level.

## Results

Table 1 describes the characteristics for migrants and non-migrants regarding the main variables used in the study. Migrants more often were younger, healthcare workers with patient contact, and had lower completed education levels, while the groups were approximately similar in terms of comorbidities. A larger percentage of the migrant respondents were male. Among migrants, 68.1% reported having been offered a vaccine, compared to 81.1% of non-migrants. The percentage of those taking the vaccine among those offered was lower among migrants as compared to non-migrants (79.9% vs. 91.1%) while there were twice as many who reported having had COVID-19 among migrants than non-migrants (8.4% vs. 4.0%).

**Table 1:** Descriptive statistics of migrants and non-migrants by main study variables

Variable	Non-migrants, n (%)	Migrants, n (%)
Sample size *(% of net sample, n* = *5442)*	3596 (66.1)	1284 (23.6)
Nordic-born	N/A	170 (13.2)
EU-born	N/A	455 (35.4)
Offered COVID-19 vaccine	2917 (81.1)	875 (68.1)
Received COVID-19 vaccine *(% of those offered)*	2657 (91.1)	699 (79.9)
Confirmed case of COVID-19	143 (4.0)	108 (8.4)
Male	1421 (39.5)	630 (49.1)
Female	2174 (60.5)	654 (50.9)
Age: 18–29	382 (10.6)	160 (12.5)
Age: 30–44	901 (25.1)	569 (44.3)
Age: 45–59	1026 (28.5)	371 (28.9)
Age: 60 +	1286 (35.8)	184 (14.3)
Any underlying health condition	939 (26.1)	333 (25.9)
Job type: non-health-related	2030 (56.5)	767 (59.7)
Job type: healthcare workers	279 (7.8)	153 (11.9)
Language spoken at home: only Norwegian	3348 (93.1)	217 (16.9)
Language spoken at home: Norwegian and another	217 (6.0)	810 (63.1)
Language spoken at home: only another	14 (0.4)	249 (19.4)
Education: no university	1358 (37.8)	519 (40.4)
Education: some university	2212 (61.5)	749 (58.3)

The first logistic regression analysis compared the outcome of being offered a vaccine based on migrant background, with successive controls for variables that were used in prioritizing vaccination roll-out in Norway and Oslo specifically (Table 2). As expected, age was significantly associated with being offered a vaccine for all participants, with increasing odds ratios (ORs) with older age. Respondents with underlying health conditions were also more likely to be offered the vaccine than those who did not report underlying health conditions. According to the prioritization strategy, healthcare workers were the only group that reported a higher OR relative to the reference category of “Other jobs without contact”. Based on these results, we collapsed this category into health-related and non-health-related jobs in subsequent analyses, in order to simplify and clarify interpretation. Increases in the Nagelkerke R square values in the models indicate that most of the variation was explained by age group and to a lesser extent job type. However, migrant background remained significant as each control variable was added. In model 4, controlling for all variables, migrants still had a significantly lower OR of 0.65 (95% CI: 0.52–0.82) relative to the non-migrant group.

**Table 2:** Logistic regression results for the outcome of being offered a COVID-19 vaccine by migrant status, controlling for covariates related to prioritization of vaccination

Covariates	N (%) Not Offered	N (%) Offered	Model 1 (Migrant)	Model 2 (Age)	Model 3 (Health)	Model 4 (Job Type)
Non-migrant	593 (25.7)	1710 (74.3)	Ref	Ref	Ref	Ref
Migrant	334 (36.3)	585 (63.7)	**.61 (.52-.72)**	.84 (.68–1.02)	**.81 (.66-.99)**	**.65 (.52-.82)**
18–29	216 (59.2)	149 (40.8)		Ref	Ref	Ref
30–44	694 (53.5)	603 (46.5)		**1.28 (1.01–1.62)**	**1.31 (1.03–1.66)**	**1.73 (1.32–2.26)**
45–59	15 (1.3)	1145 (98.7)		**110.41 (63.66–191.47)**	**108.14 (62.27–187.82)**	**158.51 (89.84–279.66)**
60 +	2 (0.5)	398 (99.5)		**283.02 (69.44–1153.60)**	**260.06 (63.75–1060.97)**	**377.34 (91.88–1549.74)**
No underlying conditions	853 (32.5)	1775 (67.5)			Ref	Ref
At least one condition	74 (12.5)	520 (87.5)			**2.62 (1.94–3.53)**	**3.03 (2.21–4.16)**
Other work without contact	106 (34.1)	205 (65.9)				Ref
Customer service with contact	151 (30.0)	353 (70.0)				1.25 (.84–1.86)
Office work with others	169 (33.7)	333 (66.3)				.79 (.53–1.19)
Office work, mostly home office	367 (33.6)	725 (66.4)				.78 (.54–1.12)
School	114 (30.0)	267 (70.0)				.76 (.49–1.17)
Health service with patient contact	20 (4.6)	412 (95.4)				**16.33 (9.32–28.60)**
**N**	927 (28.8)	2295 (71.2)				
**Model sig**			.000	.000	.000	.000
**-2 log likelihood**			3831.905	2467.601	2425.389	2166.631
**Nagelkerke R square**			.015	.504	.516	.587

We subsequently conducted regression models controlling for different socioeconomic and demographic characteristics, including sex, and highest completed education level (Table 3). Migrants still had a significantly lower OR for being offered the vaccine relative to non-migrant respondents (Model 3 OR 0.49, 95% CI: 0.42–0.57). In the full model, females were more likely than males to report being offered the vaccine, while individuals with some university education were less likely to do so relative to those with no university education. While the models improve with each included covariate, the overall explanatory strength remains relatively low, and the ORs for migrants remain similar for the successive models.

**Table 3:** Logistic regression results for the outcome of being offered a COVID-19 vaccine by migrant status, controlling for demographic and socioeconomic covariates

Variable	N (%) Not Offered	N (%) Offered	Model 1 (Migrant)	Model 2 (Sex)	Model 3 (Education)
Non-migrant	677 (19.0)	2892 (81.0)	Ref	Ref	Ref
Migrant	404 (31.9)	864 (68.1)	**.50 (.43-.58)**	**.51 (.44-.59)**	**.49 (.42-.57)**
Male	495 (24.3)	1541 (75.7)		Ref	Ref
Female	586 (20.9)	2215 (79.1)		**1.15 (1.00–1.32)**	**1.20 (1.04–1.38)**
No university	285 (15.2)	1591 (84.8)			Ref
Some university	796 (26.9)	2165 (73.1)			**.47 (.40-.54)**
**N**	1081 (22.3)	3756 (77.7)			
**Model sig**			**.000**	**.000**	**.000**
**-2 log likelihood**			**5054.515**	**5050.583**	**4947.007**
**Nagelkerke R square**			**.027**	**.028**	**.060**

The next analyses were restricted to the migrant subsample only, so comparisons could be made based on birth country, years in Norway and language spoken at home. Table 4 shows the results of the full models for separate comparisons of Nordic vs. non-Nordic-born, EU vs non-EU-born, and the top five represented birth countries: Pakistan, Philippines, Poland, Somalia and Sweden. Controlling for the abovementioned variables, there were no significant differences comparing Nordic- vs. non-Nordic-born or EU vs. non-EU-born respondents. Only respondents born in the Philippines had a significantly higher OR of being offered the vaccine, relative to the reference group of Swedish-born respondents, although the lower OR for Pakistan had a p-value of 0.058. Additionally, the only control variable to show a significant difference despite the classification of migrants was the number of years lived in Norway, with those living in the country fewer than 15 years having lower ORs of 0.22 for all comparisons. For comparisons based on migrants from the top five countries, a lower OR for speaking only a foreign language at home suggest an association with lower reported vaccine offer although the variable as a whole was non-significant.

**Table 4:** Logistic regression results for the outcome of being offered a COVID-19 vaccine by birth country (migrants only) classified into three different ways, controlling for years lived in Norway and language spoken at home

Covariate	N (%) Not Offered	N (%) Offered	Full Model	Covariate	N (%) Not Offered	N (%) Offered	Full Model	Covariate	N (%) Not Offered	N (%) Offered	Full Model
Born Nordic	33 (20.0)	132 (80.0)	Ref	Born EU	156 (35.4)	285 (64.6)	Ref	Sweden	20 (22.2)	70 (77.8)	Ref
Born non-Nordic	347 (34.0)	674 (66.0)	.70 (.46–1.05)	Born non-EU	224 (30.1)	521 (69.9)	1.18 (.91–1.52)	Pakistan	25 (36.2)	44 (63.8)	.50 (.24–1.02)
Median (15 years) or longer in Norway	88 (14.3)	527 (85.7)	Ref	Median (15 years) or longer in Norway	88 (14.3)	527 (85.7)	Ref	Philippines	8 (16.7)	40 (83.3)	**3.24 (1.32–7.91)**
Fewer years than median in Norway	292 (51.1)	279 (48.9)	**.22 (.17-.29)**	Fewer years than median in Norway	292 (51.1)	279 (48.9)	**.22 (.17-.29)**	Poland	33 (50.0)	33 (50.0)	.80 (.38–1.71)
Only Norwegian	37 (17.7)	172 (82.3)	Ref	Only Norwegian	37 (17.7)	172 (82.3)	Ref	Somalia	15 (34.1)	29 (65.9)	.57 (.25–1.29)
Norwegian and other	233 (31.4)	509 (68.6)	.96 (.64–1.44)	Norwegian and other	233 (31.4)	509 (68.6)	.88 (.59–1.32)	Median (15 years) or longer in Norway	27 (16.0)	142 (84.0)	Ref
Only other	110 (46.8)	125 (53.2)	.75 (.47–1.19)	Only other	110 (46.8)	125 (53.2)	.70 (.44–1.12)	Fewer years than median in Norway	74 (50.0)	74 (50.0)	**.22 (.12-.38)**
								Only Norwegian	4 (8.2)	45 (91.8)	Ref ^1^
								Norwegian and other	66 (31.1)	146 (68.9)	.47 (.18–1.25)
								Only other	31 (55.4)	25 (44.6)	**.31 (.10-.93)**
**N**	380 (32.0)	806 (68.0)		**N**	380 (32.0)	806 (68.0)		**N**	101 (31.9)	216 (68.1)	
**Model sig**			**.000**	**Model sig**			**.000**	**Model sig**			**.000**
**-2 log likelihood**			**1516.976**	**-2 log likelihood**			**1518.434**	**-2 log likelihood**			**396.837**
**Nagelkerke R square**			**.174**	**Nagelkerke R square**			**.173**	**Nagelkerke R square**			**.240**

We ran additional regression models combining the vaccine prioritization variables with years lived in Norway for migrants born in the top five represented countries informed by results from the previous analyses (Table 5). In the fully adjusted model (model 5), none of the individual countries remained statistically significant compared to Sweden, although results for Poland (lower OR) were significant in models 1–4.The variable as a whole was not significant in model 4, however, but results also were suggestive for the Philippines having a higher OR and Somalia and Pakistan having lower ORs than the Sweden reference group, despite not reaching statistical significance. Older ages were still more likely to have received a vaccination offer, although the oldest age group was not significant, likely due to the small sample size. Additionally, there were still higher ORs for those with underlying health conditions and for healthcare workers with patient contact, and a lower OR for migrants who had lived in Norway fewer than the median years.

**Table 5:** Logistic regression results for the outcome of being offered a COVID-19 vaccine by birth country (migrants only), controlling for covariates related to prioritization of vaccination and years lived in Norway

Covariate	N (%) Not Offered	N (%) Offered	Model 1 (Birth Country)	Model 2 (Age)	Model 3 (Health)	Model 4 (Job Type)	Model 5 (Years in Norway)
Sweden	19 (26.8)	52 (73.2)	Ref	Ref	Ref	Ref	Ref
Pakistan	13 (31.7)	28 (68.3)	.79 (.34–1.83)	.58 (.22–1.52)	.53 (.19–1.44)	.44 (.15–1.30)	.34 (.11–1.10)
Philippines	7 (20.0)	28 (80.0)	1.46 (.55–3.90)	2.12 (.73–6.12)	2.26 (.78–6.58)	.89 (.25–3.11)	1.28 (.34–4.74)
Poland	28 (50.0)	28 (50.0)	**.36 (.17-.77)**	**.37 (.16-.86)**	**.34 (.15-.81)**	**.36 (.14-.89)**	.46 (.18–1.19)
Somalia	11 (35.5)	20 (64.5)	.66 (.27–1.64)	.58 (.21–1.65)	.64 (.22–1.83)	.53 (.17–1.66)	.38 (.12–1.28)
18–29	12 (54.5)	10 (45.5)		Ref	Ref	Ref	Ref
30–44	62 (47.7)	68 (52.3)		1.67 (.63–4.44)	1.85 (.67–5.07)	1.65 (.56–4.88)	1.74 (.57–5.35)
45–59	3 (4.3)	67 (95.7)		**36.64 (8.26–162.58)**	**35.84 (7.89–162.85**	**40.02 (8.37–191.39)**	**30.29 (6.07–151.10)**
60 +	1 (8.3)	11 (91.7)		**19.05 (1.96–185.04)**	**15.30 (1.51–155.34)**	8.33 (.72–95.90)	7.43 (.60–92.52)
No underlying condition	72 (38.5)	115 (61.5)			Ref	Ref	Ref
Any underlying condition	6 (12.8)	41 (87.2)			**3.52 (1.26–9.88)**	**4.32 (1.50–12.45)**	**4.29 (1.47–12.48)**
Non-health job	74 (41.3)	105 (58.7)				Ref	Ref
Health job	4 (7.3)	51 (92.7)				**12.94 (4.03–41.50)**	**12.80 (3.96–41.33)**
Median (15 years) or longer in Norway	23 (19.2)	97 (80.8)					Ref
Fewer years than median in Norway	55 (48.2)	59 (51.8)					**.38 (.16-.91)**
**N**	78 (33.3)	156 (66.7)					
**Model sig**			**.024**	**.000**	**.000**	**.000**	**.000**
**-2 log likelihood**			**286.688**	**228.929**	**222.464**	**195.953**	**191.137**
**Nagelkerke R square**			**.065**	**.354**	**.383**	**.490**	**.509**

Finally, in order to more closely investigate the role of time lived in Norway, we ran models for different combinations of comparisons between non-migrant respondents and/or migrant groups categorized by length of stay in Norway. Controlling for the prioritization variables, migrants who have lived in Norway for fewer years than the median had significantly lower ORs of being offered a vaccine compared to non-migrant respondents (OR 0.59, 95% CI: 0.45–0.77) and migrants who have lived in Norway for the median or longer (OR 0.51, 95% CI: 0.34–0.74), respectively. The migrants who have lived in Norway for the median or longer have similar odds relative to non-migrant respondents. As expected, the vaccination prioritization variables were also significant predictors of being offered a vaccine, showing similar trends as in previous analyses (Table 6).

**Table 6:** Logistic regression results for the outcome of being offered a COVID-19 vaccine by years lived in Norway, controlling for covariates related to prioritization of vaccination

Covariate	N (%) Not Offered	N (%) Offered	Full Model	Covariate	N (%) Not Offered	N (%) Offered	Full Model	Covariate	N (%) Not Offered	N (%) Offered	Full Model
Non-migrant	593 (25.7)	1710 (74.3)	Ref	Non-migrant	593 (25.7)	1710 (74.3)	Ref	Migrant, lived in Norway 15 + years	80 (19.0)	340 (81.0)	Ref
Migrant, lived in Norway < 15 years	237 (52.7)	213 (47.3)	**.59 (.45-.77)**	Migrant, lived in Norway 15 + years	80 (19.0)	340 (81.0)	1.08 (.76–1.54)	Migrant, lived in Norway < 15 years	237 (52.7)	213 (47.3)	**.51 (.34-.74)**
18–29	197 (58.8)	138 (41.2)	Ref	18–29	167 (57.8)	122 (42.2)	Ref	18–29	62 (64.6)	34 (35.4)	Ref
30–44	620 (54.9)	509 (45.1)	**1.54 (1.16–2.04)**	30–44	496 (51.1)	475 (48.9)	**1.78 (1.31–2.42)**	30–44	246 (54.4)	206 (45.6)	1.57 (.94–2.61)
45–59	12 (1.3)	929 (98.7	**141.41 (75.72–264.12)**	45–59	10 (0.9)	1069 (99.1)	**207.99 (105.03–411.85)**	45–59	8 (3.0)	258 (97.0)	**50.73 (21.48–119.80)**
60 +	1 (0.3)	347 (99.7)	**596.48 (82.34–4320.89)**	60 +	0 (0.0)	384 (100.0)	-	60 +	1 (1.8)	55 (98.2)	**73.89 (9.54–572.46)**
No underlying	765 (33.7)	1506 (66.3)	Ref	No underlying	630 (28.4)	1588 (71.6)	Ref	No underlying	285 (40.9)	412 (591)	Ref
Any underlying	65 (135)	417 (76.5)	**2.89 (2.06–4.06)**	Any underlying	43 (8.5)	462 (91.5)	**3.77 (2.57–5.55)**	Any underlying	32 (18.5)	141 (81.5)	**2.43 (1.46–4.04)**
Non-health job	818 (33.8)	1601 (66.2)	Ref	Non-health job	661 (28.0)	1697 (72.0)	Ref	Non-health job	307 (42.1)	422 (57.9)	Ref
Health job	12 (3.6)	322 (96.4)	**25.85 (14.13–47.28)**	Health job	12 (3.3)	353 (96.7)	**21.71 (11.78–39.99)**	Health job	10 (7.1)	131 (92.9)	**13.08 (6.53–26.22)**
**N**	830 (30.1)	1923 (69.9)		**N**	673 (24.7)	2050 (75.3)		**N**	317 (36.5)	553 (63.6)	
**Model sig**			**.000**	**Model sig**			**.000**	**Model sig**			**.000**
**-2 log likelihood**			**1876.230**	**-2 log likelihood**			**1618.109**	**-2 log likelihood**			**720.863**
**Nagelkerke R square**			**.593**	**Nagelkerke R square**			**.606**	**Nagelkerke R square**			**.524**

## Discussion

To the best of our knowledge, our study is the first to show that migrants report a significantly lower probability, by approximately a third, of being offered the vaccine than non-migrants. The main factors taken into account by Norway when initially offering the vaccine were age, having comorbidities, working in a health care, and, later, living in an area with high rates of infection. While there were substantially fewer migrants in the oldest age category, there was an approximately even share of migrants and non-migrants among those reporting underlying health conditions in our study population. Further, a larger proportion of migrants than non-migrants worked in healthcare jobs with patient contact. All the parishes in our survey were among the prioritised areas for vaccination and had a high percentage of migrants, which allowed us to recruit many migrants. Nonetheless, even after controlling for the prioritization variables, which should theoretically have been the only contributors to observed disparities, significant differences between migrants and non-migrants were still observed.

However, differences in receiving an offer cannot fully explain the disparities in vaccination uptake. Unadjusted survey results show lower vaccination rates for migrants as compared to non-migrants among those offered a vaccine (79.9% vs. 91.1%), which is consistent with register data from Norway [9] and research in other countries regarding low vaccination uptake among migrants and ethnic minorities [21]. Nonetheless, our results suggest that issues associated with the necessary precondition of being offered a vaccine also play a role in observed vaccination differences. However, we cannot take for granted that being offered a vaccine is the same as reporting the offer, as the offer must be recognized and understood as such by the recipient to be reported. In-depth exploration of this process would require a qualitative approach, but our data can give some light to the mechanisms underlying the process.

Analyses of demographic and socioeconomic factors indicate higher level of completed education and female sex as, respectively, negatively and positively associated with reporting an offer of the vaccine. Females more often use health services and are therefore to a higher degree registered with the GP with several health conditions. Higher educated people tend to be healthier and use the GP to a lesser degree. Correlations with other variables that we have not measured, could also partially explain why people with higher levels of completed education reported having been offered a vaccine to a lesser degree. Further study, including interactions with income and other related variables, is necessary, as these results might reflect an underlying mechanism increasing health inequalities, especially since low income has previously been correlated with both notified cases of COVID-19 and hospitalizations in Norway [22].

To better understand the underlying mechanisms in the differences in access specific to migrants, we considered two variables related to the migrant background of the participants: language spoken at home and length of stay in the country. Language has previously been understood as the main key to vaccine uptake among migrants, and translating information has been the main strategy in the Nordic countries to target this group [23]. However, in our analyses, language spoken at home was not significantly related to reporting a vaccine offer among migrants. Similarly, there is growing evidence from Norway and Sweden showing higher rates of disease and death among non-migrants married to migrants compared to non-migrant only couples, supporting the suggestion that language alone is insufficient for explaining higher hospitalization and COVID-19 death rates among migrants [24, 25]. However, our question did not directly assess Norwegian proficiency for the individual participant, which might have diluted any association if some of the participants who spoke both or only other languages at home were proficient in Norwegian. Supporting this is the fact that we could find some association between vaccine offer acknowledgment and those who only spoke a foreign language at home in the analyses by country of origin. Also, a strongly contributing reason for our results might be the self-selection of participants in this survey, as more than 90% of respondents completed the survey in Norwegian despite having the option to complete the survey in different languages.

The duration of residence in Norway emerged as a key and consistent explanatory variable for vaccine offer, in accordance with a recent Norwegian report of actual vaccination rates among migrants [9]. The length of time a migrant has lived in a country is a complex variable related to several possible bottlenecks in the offer of the vaccination as shown in Fig. 1, such as inclusion in the municipal population register where the individuals have registered their age and address; access to and use of health care services, including GPs who were key in providing information on comorbidities [15]; and the degree of health literacy and digital literacy related to receiving the message and understanding it in Norwegian, which has recently been shown to be lower among migrants [26, 27]. Thus, all these factors should be specifically addressed in the future by policymakers and health services organizers when trying to reach the whole population with vaccination programs or other health programs.

We detected very few significant differences in being offered the vaccine based on birth country when we compared countries to each other. For comparisons of the top five represented countries, Sweden was selected as the reference for logistic regression because of its proximity and linguistic and cultural similarities to Norway. However, due to these factors and possible family and work connections leading to frequent mobility between the two at least when travel/borders were relatively open, many Swedish-born migrants may be registered in Sweden and have been offered the vaccine through the Swedish system. If so, their experiences may not fully reflect those of either the Norwegian-born group or people born in other countries, leading to confounding of results for these comparisons. The number of individuals in the other national groups were probably too low to be able to find statistically significant results at the 0.05 level. However, in addition to significantly lower ORs for migrants from Poland, interesting trends in these analyses are the tendencies of lower ORs for reporting being offered a vaccine for migrants from Pakistan and Somalia and the high OR for those from the Philippines, which mirror the vaccination rates of these groups in the national statistics [9]. This happened even though in Oslo municipality, where this study was conducted, Polish and Somali speaking personal were at times available and responsible for making invitations by phone to those with names that indicated origin from those areas.

Our sampling method targeted the six eastern parishes in Oslo where the largest shares of migrants live in Norway. Migrants are generally younger than non-migrants, more often have health care related jobs and have been more often infected by COVID-19 [9]. In these ways, the differences among migrants and non-migrants in our population are as expected. Although we cannot explain the reasons for a higher representation of men in the migrant population, our results are adjusted for sex. Even though one of the strengths of this study is the high percentage of migrants in our survey, including nearly a fourth of the net sample, they are still underrepresented relative to the areas we chose and, as stated above, the numbers might have been too small to detect significant differences for some analyses. Furthermore, the composition of countries and the long mean length of stay reveals that migrants in our survey were not representative of the migrant population in Norway [28]. Thus, even though we adjusted for several socioeconomic, epidemiological, and other factors, these analyses must be cautiously interpreted. Additional weaknesses with our survey are the generally low response rate which is otherwise typical for this type of survey, and that the population reached was composed of persons that are registered in the municipality. Thus, people who could not be reached to get an offer of the vaccine because of lack of registration, were not included in our population either. Probably, the share of migrants reporting having received an invitation to be vaccinated had been even lower if we had reached those with shorter lengths of stay in Norway and those with lower Norwegian proficiency levels.

Finally, it could be argued that our results do not reflect the actual offer of a vaccination but rather the report of a perceived offer by the population. It is possible that respondents did not recognize an offer or do not recall receiving it. It is known, for example, that migrants who do not speak Norwegian do not answer the telephone when the caller is not known. However, as shown in Fig. 1 and stated in the framework by Levesque et al., the perception of an offer is necessary to be able to seek, reach, and engage in healthcare. From that point of view, equity in health care services is not determined by the sender of a health care message, but by the receiver.

## Conclusion

In a pandemic, nobody is safe before all are safe. Understanding the complex causes of lower vaccination rates among migrants is key to improving public health. Our results point to structural reasons that may partly explain lower rates of vaccination among migrants. We suggest different mechanisms that should be addressed to improve health for all in a pandemic situation and point to length of stay in Norway as a complex key variable to explain differences that should be further studied.

## Supplementary Information

**Additional file 1:** **Supplementary Table 1. **Survey questions usedin analyses. **Supplementary Table 2.** Net response rates, composition of and differencesbetween the net and gross samples.

## Data Availability

The datasets used and analysed during the current study are available from the corresponding author on reasonable request.

## Abbreviations

GP: General Practitioner
OR: Odds ratio
