# Supplementary material for: Disparities in the offer of COVID-19 vaccination to migrants and non-migrants in Norway: a cross sectional survey study
Source: BMC Public Health. 2022 Jul 4;22:1288. doi: 10.1186/s12889-022-13687-8 (PMC9252073; doi:10.1186/s12889-022-13687-8)
Supplement: Supplementary file 1 — Additional file 1: Supplementary Table 1. Survey questions usedin analyses. Supplementary Table 2. Net response rates, composition of and differencesbetween the net and gross samples. [file 12889_2022_13687_MOESM1_ESM.docx]

**Supplementary Table 1.** Survey questions used in analyses.

| **Question** | **Possible Answers in Survey** | **Modifications for Analyses** |
| --- | --- | --- |
| Have you been offered a vaccine against COVID-19? | Yes or no  If yes: Have you received the vaccine? Yes or no | N/A |
| Have you experienced the following in connection with the outbreak of the coronavirus (COVID-19)? | Possible answers quarantine, isolation, probable case, confirmed (diagnosed) case, hospitalization, as well as whether others in the household had a confirmed or were hospitalized, and none of these | We only used conformed diagnosed case for the analyses |
| In what country were you born? (Only asked of those born outside Norway) | 63 countries, as well as “Another country” and “Do not want to answer” | Responses were grouped as: a) Nordic (excluding Norway; Greenland and Åland Islands were not listed as options) vs. non-Nordic (all other options), and b) EU (includes EEA, Switzerland, and Great Britain. Republic of Cyrus was not listed as an option, and Norway excluded) vs. non-EU (all other options). Further, the top 5 most represented countries were identified, excluding “Another country”, which was the most represented overall. |
| How long have you lived in Norway? (Only asked of those born outside Norway) | Open text box for number of years | The median was calculated as 15 years. Respondents who answered were categorized as below the median or at the median or above. |
| Sex | Used information from Kantar database; only male and female possible. | N/A |
| Age Group | Used information from Kantar database. | Grouped in categories of 18-29, 30-44, 45-59, and 60+ |
| Some groups may be at higher risk for severe COVID-19. Do you have? | Allowed to select yes/no for: chronic lung disease; chronic cardiovascular disease; liver failure; kidney failure; neurological disease or injury; impaired immune system confirmed by a doctor or hospital; diabetes; mental or other learning or developmental disabilities; impaired hearing or vision, blind or deaf; physical mobility impairment. Most options included clarifying examples. | Classified into two categories: no underlying conditions and at least one underlying condition. Excluded respondents who did not answer yes or no to most or all of the categories. |
| What kind of job do you have? Select the option that fits best / you spend the most time on. [During the pandemic]. | Customer service representative who must have personal contact; Office work in an office community with others; Office work where you can mostly work at home; Work in educational institution/school/ kindergarten etc. with contact with children/youth; Work in health trusts with patient contact; Other work without contact with customers/patients/ children and young people. | Initial analyses use all categories, while some secondary analyses collapse the responses into health-related or non-health jobs. |
| What language do you speak at home? (Asked of all respondents regardless of birth country) | a) Only Norwegian, b) Norwegian and another language, c) Only a language other than Norwegian | N/A |
| What is your highest completed schooling? | Primary school education - 10-year primary school, 7-year primary school; Upper secondary general education; Upper secondary vocational education; Vocational school/ Vocational education (1/2 - 2 years) based on upper secondary vocational education; University/college education with up to 4 years duration; University/college education with more than 4 years duration | Collapsed into at least some university (the last two options) and no university (remaining options) |

**Supplementary Table 2.** Net response rates, composition of and differences between the net and gross samples

|  | Net response rates (%) | Composition of gross sample n (%) | Composition of net sample n (%) | Percentage points differences between net and gross samples |
| --- | --- | --- | --- | --- |
| Total | 9.1 | 59978 (100) | 5442 (100) |  |
| Sex |  |  |  |  |
| Male | 7.4 | 30764 (51.3) | 2270 (41.7) | -9.6 |
| Female | 10.9 | 29214 (48.7) | 3171 (58.3) | 9.6 |
| Age |  |  |  |  |
| 18-29 | 6.8 | 10849 (18.1) | 737 (13.5) | -4.6 |
| 30-44 | 8.7 | 18781 (31.3) | 1637 (30.1) | -1.2 |
| 45-59 | 9.8 | 15267 (25.5) | 1496 (27.5) | 2.0 |
| 60+ | 10.4 | 15081 (25.1) | 1571 (28.9) | 3.8 |
| Parish |  |  |  |  |
| Alna | 8.1 | 12222 (20.4) | 987 (18.1) | -2.3 |
| Bjerke | 9.2 | 8384 (14.0) | 772 (14.2) | 0.2 |
| Gamle Oslo | 11.1 | 14761 (24.6) | 1637 (30.1) | 5.5 |
| Grorud | 8.0 | 6768 (11.3) | 538 (9.9) | -1.4 |
| Stovner | 7.4 | 8204 (13.7) | 611 (11.2) | -2.5 |
| Søndre Nordstrand | 9.3 | 9639 (16.1) | 896 (16.5) | 0.4 |
